# Prognostic Factors in Severe Chagasic Heart Failure

**DOI:** 10.5935/abc.20170027

**Published:** 2017-03

**Authors:** Sandra de Araújo Costa, Salvador Rassi, Elis Marra da Madeira Freitas, Natália da Silva Gutierrez, Fabiana Miranda Boaventura, Larissa Pereira da Costa Sampaio, João Bastista Masson Silva

**Affiliations:** Universidade Federal de Goiás - (UFG), Goiânia, GO - Brazil

**Keywords:** Heart Failure / mortality, Prognosis, Chagas Cardiomyopathy, Chagas Disease

## Abstract

**Background:**

Prognostic factors are extensively studied in heart failure; however, their role in severe Chagasic heart failure have not been established.

**Objectives:**

To identify the association of clinical and laboratory factors with the prognosis of severe Chagasic heart failure, as well as the association of these factors with mortality and survival in a 7.5-year follow-up.

**Methods:**

60 patients with severe Chagasic heart failure were evaluated regarding the following variables: age, blood pressure, ejection fraction, serum sodium, creatinine, 6-minute walk test, non-sustained ventricular tachycardia, QRS width, indexed left atrial volume, and functional class.

**Results:**

53 (88.3%) patients died during follow-up, and 7 (11.7%) remained alive. Cumulative overall survival probability was approximately 11%. Non-sustained ventricular tachycardia (HR = 2.11; 95% CI: 1.04 - 4.31; p<0.05) and indexed left atrial volume ≥ 72 mL/m^2^ (HR = 3.51; 95% CI: 1.63 - 7.52; p<0.05) were the only variables that remained as independent predictors of mortality.

**Conclusions:**

The presence of non-sustained ventricular tachycardia on Holter and indexed left atrial volume > 72 mL/m^2^ are independent predictors of mortality in severe Chagasic heart failure, with cumulative survival probability of only 11% in 7.5 years.

## Introduction

Heart failure (HF) is a clinical syndrome in which the heart cannot provide a cardiac output that meets the needs of the peripheral organs and tissues, or does it under conditions of high filling pressures in its chambers.^1^

The American Heart Association (AHA) estimates a HF prevalence of 5.1 million individuals in the United States between 2007 and 2012.^2^ In Brazil, the HF prevalence is 2 million patients, and its incidence, 240,000 new cases per year.^3^

Chagas disease is still an important etiology of HF. Approximately 10-12 million people worldwide are infected with *Tripanossoma cruzi*, and 21% to 31% of them will develop cardiomyopathy. This pathology accounts for 15,000 deaths per year and approximately 200,000 new cases. In Brazil, there are 3 million people with Chagas disease.^1^

Knowledge and experience indicate that the prognosis of individuals with HF is poor, and, of all etiologies, Chagasic HF has the worst prognosis.^4^

Studies on the poor prognosis of patients with Chagasic HF have been valued. However, information on mortality predictors in that disease are limited, and knowing those factors enables the treatment in the presence of some unfavorable conditions.^5-7^

Access to those parameters is usually easy, inexpensive and allows identifying the patients at higher mortality risk.

This study was aimed at identifying the association of clinical and laboratory factors with the prognosis of severe Chagasic HF, as well as the association of those factors with mortality rate and survival in a 7.5-year follow-up.

## Methods

This is a subset of the "*Estudo Multicêntrico, Randomizado de Terapia Celular em Cardiopatias (EMRTCC) - Cardiopatia Chagásica*", with retrospective analysis of prospectively collected data.^8^

The research was conducted at the Heart Failure Service of the Hospital das Clínicas (HC) of the Goiás Federal University (UFG).

This study's target population was formed by 60 patients of the 234 participants in the EMRTCC, who remained being followed up at the HF outpatient clinic of the HC/UFG.

The EMRTCC study showed that the intracoronary injection of autologous stem-cells conferred no additional benefit over standard therapy to patients with Chagasic cardiomyopathy. Neither the left ventricular function nor the quality of life of those patients improved.^9^ The neutral result ensured that the population assessed had no interference of that procedure.

The complete follow-up duration in this study was 7.5 years.

### Analyzed parameters

#### Systolic blood pressure

Systolic blood pressure (SBP) was measured by using the auscultatory technique standardized by the VI Brazilian Guidelines on Arterial Hypertension, with duly calibrated aneroid sphygmomanometer and stethoscope. Normality was considered as SBP of 120 mm Hg and diastolic blood pressure (DBP) of 80 mmHg.^10^

#### Age

Age was calculated based on the birth date recorded on the patient's identification document, considering the complete years of life at the time of study selection.

#### Simpson's left ventricular ejection fraction

Left ventricular ejection fraction (LVEF) was measured by echocardiography with the Simpson's method. All exams were performed by one single examiner in a Toshiba Xario device.

#### Serum sodium

Ion-selective electrode photometry was used to measure serum sodium concentration.^11^ The normal reference value adopted at the local analysis laboratory was 135-144 mEq/L. Serum sodium concentration below the lower limit of normality (< 135 mEq/L) was considered hyponatremia, and above 144 mEq/L, hypernatremia.^11^

#### Creatinine

Automated Jaffe's reaction was used to measure serum creatinine concentration. The reference values adopted for creatinine were 0.7 - 1.3 mg/dL for men, and 0.6 - 1.2 mg/dL for women.^11^

#### 6-minute walk test

The 6-minute walk test (6MWT) was performed twice, at a minimum 15-minute interval for rest. At the end of the 6MWT, the vital data initially obtained were collected again, and the distance covered by the patient was calculated as the mean of the two tests.^12^

The normal reference values for the 6MWT ranged from 400m to 700m for healthy individuals. So far, the literature has no standardized 6MWT reference value for individuals with heart disease.^13^ We adopted the value of ≥ 400m for a satisfactory result, and < 400m for an unsatisfactory result, based on data published in the SOLVD Study.^14^

#### Non-sustained ventricular tachycardia

Non-sustained ventricular tachycardia (NSVT) was defined as three or more consecutive heartbeats, originating below the atrioventricular node, with heart rate > 100 beats per minute and duration < 30 seconds, identified on 24-hour Holter.^15^

#### QRS width

The QRS width was obtained on an electrocardiographic tracing in a duly calibrated device. Values ≤ 120ms were considered normal QRS width, while those > 120ms, extended QRS.^16^

#### Indexed left atrial volume

Indexed left atrial volume (ILAV) was obtained from the left atrial contour in two orthogonal views (apical 2- and 4-chamber views)^17^ on echocardiography performed by a single observer in all patients.

Values up to 34 mL/m^2^ were considered normal, between 35 and 41 mL/m^2^, mild increase, between 42 and 48 mL/m^2^, moderate increase, and greater than 48 mL/m^2^, significant increase.^17^

#### Functional class

Functional class was categorized based on the New York Heart Association (NYHA) classification, whose validity and reliability have been well established.^14^ The classification was based on the severity of the symptoms reported, and ranged from I to IV.^14^

#### Statistical analysis

The data were collected and recorded in an electronic spreadsheet and analyzed with the IBM SPSS statistical software, version 21.0.

The categorical variables were expressed as frequency, with absolute numbers and proportions. The association analysis between predicting variables and outcomes was performed with the chi-square test.

The chi-square test was used to compare outcome (death) and the different categories of predicting variables, such as age group, SBP, serum sodium, NSVT and QRS width.

The continuous quantitative variables were expressed as means, medians (non- parametric distribution), standard deviation and confidence interval (CI). Data distribution was analyzed by using the Shapiro Wilks test, considering the sample size smaller than 100 participants. To compare the means of the predicting variables, non-paired Student *t* test or Mann Whitney *U* test was used, depending on data distribution.

All tests were performed considering the 5% significance level, two-tailed probability and 95% CI.

#### Survival analysis

The survival time was calculated as the interval between the dates of treatment beginning and death. The maximal follow-up duration was 90 months, and those remaining alive after that time were censored. Because the participants underwent different follow-up durations and entered the study at different times, their prognoses were assessed with Kaplan-Meier statistics.

To compare stratified survival curves, hazard ratio (HR) was used as the measure of association between survival variables. Log-Rank (Mantel-Cox) test was used to compare the expected values of each stratum under the null hypothesis that the risk is the same in all strata, that is, the number of events observed in each category of the variable analyzed, with the number of events (outcomes) expected.

Cox proportional hazards model, a semiparametric model to estimate the proportionality of hazards during the entire follow-up in an adjusted way, was performed to estimate the effect of the predicting variables. The continuous variables whose p-value < 0.20, in their quantitative format, and the categorical dichotomous or polychotomous variables were included in the model. The p-value of the Wald test was used.

Initially, univariate analysis of risk estimation was performed, and only the variables showing association with p < 0.20 were entered in the multivariate model. The model was adjusted step-by-step, with the inclusion of the variable that associated best in the first step, and considering theoretical criteria of previous knowledge.

## Results

### Baseline characteristics

Table 1 shows the initial characteristics of the 60 participants in this study.

**Table 1:** Characteristics of the sample according to the variables analyzed

Variables	Mean Median	SD/ 95%CI
Age (years)	52.6 54.0	±9.4 50.2 - 55.0
Systolic blood pressure (mm Hg)	98.4 100.0	±14.2 94.8 - 102.1
LV ejection fraction (%)	27.1 26.5	±5.5 25.3 - 28.9
Serum sodium (mEq/L)	137.3 137.0	±4.2 136.2 - 138.4
Creatinine (mg/dL)	1.2 1.2	±0.3 1.1 - 1.3
6-minute walk test (meters)	433.4 433.5	±139.1 397.5 - 469.4
QRS width (ms)	125.3 120.0	±29.4 117.7 - 132.9
ILAV (mL/m^2^)	107.0 102.7	±47.8 94.7 - 119.4

SD: standard deviation; CI: confidence interval; LV: left ventricular; ms: millisecond; ILAV: indexed left atrial volume.

### Follow-up

The patients were followed up regularly at the HF outpatient clinic of the HC/UFG.

All patients were assessed at time zero and every 15 days, up to completing 60 days. This period was necessary to optimize medication for HF therapy and clinical stabilization of patients. Then, there was a baseline assessment, in which data were collected for analysis.

The patients were followed up with regular visits at 15 days, 1, 2, 4, 6, 9 and 12 months, and then every 6 months after the 1-year visit, until the end of the 7.5-year follow-up.

### Medicamentous treatment

All participants were duly medicated, according to the III Brazilian Guideline on Chronic Heart Failure and patients' tolerance to medications.^1^

Appropriate medicamentous treatment was based on the association of a loop diuretic (furosemide), an angiotensin-converting-enzyme inhibitor (ACEI - enalapril), spironolactone and a beta-blocker (carvedilol). Patients would not receive a beta-blocker in case of intolerance. Digoxin was added when the patient remained symptomatic despite the use of those drugs. An angiotensin-receptor blocker (losartan) was prescribed in case of ACEI intolerance. Amiodarone was used in patients with symptomatic ventricular arrhythmia, documented on ECG or Holter. All patients with atrial fibrillation were anticoagulated, aiming at reaching an international normalized ratio (INR) between 2.0 and 3.0.^1^

The mean doses of ACEI and beta-blocker used were 10 mg/day and 25 mg/day, respectively. We aimed at the best drug treatment for all patients, with maximum tolerated doses of each medication. This process lasted, on average, 60 days.

### Characterization of the sample according to the variables analyzed and outcome

Analyzing the clinical variables and comparing with death and non-death, the following three variables were found to be related to the mortality outcome: serum sodium, serum creatinine and ILAV.

Mean serum sodium concentrations were significantly lower in the patients who died, while mean serum creatinine levels were higher for the same outcome.

Similarly to creatinine, the mean ILAV levels were higher in patients who died.

### Survival analysis

Of the 60 participants in this study, 53 (88.3%) died during the entire follow-up (90 months), and 7 (11.7%) were censored (alive by the end of follow-up) (Table 2).

**Table 2:** Cumulative overall survival probability (Kaplan-Meier)

Time [months (year)]	Participants at risk	Cumulative survival (%)	Deaths in the time interval	Alive at the beginning of the time interval
0	60	-	-	60
12 (1 year)	42	70	18	42
24 (2 years)	30	50	12	30
36 (3 years)	28	46	2	28
48 (4 years)	24	40	4	24
60 (5 years)	14	23	10	14
72 (6 years)	10	16	4	10
84 (7 years)	8	13	2	8
90 (7.5 years)	7	11	1	7

**Figure 1:**
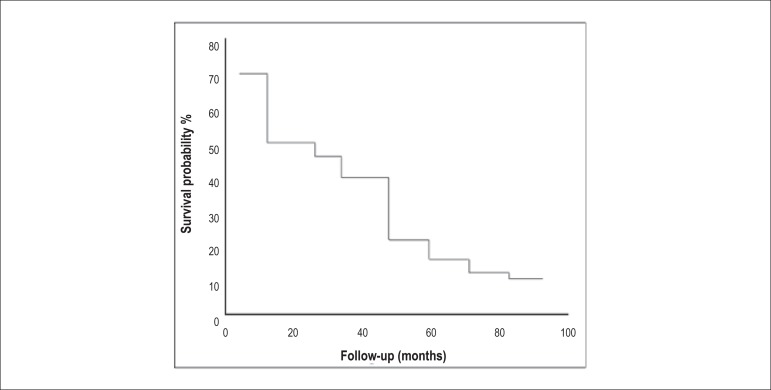
Cumulative overall survival curve.

The median follow-up was 24.5 months (±27.3; 95% CI: 28.5 - 42.6) and the cumulative overall survival probability for that follow-up period was approximately 50% (Figure 1). In the median follow-up period (24.5 months), there were 30 deaths, representing 50% of the total sample.

Most deaths were related to cardiovascular diseases, 47 (88.69%) being due to progressive HF, 3 (5.67%) to sudden death, and 1 (1.88%) to acute myocardial infarction. Of the other 2 deaths, 1 (1.88%) was due to non-Hodgkin's lymphoma, and the other (1.88%) to multiple organ failure consequent to sepsis.

### Result of the Log-Hank (Mantel-Cox) - Kaplan-Meier test

The Log-Hank (Mantel-Cox) - Kaplan-Meier test was used to compare the survival curve with general mortality for the clinical and laboratory variables.

Regarding survival, the NSVT and ILAV variables showed significance. Patients with ILAV < 72 mL/m^2^ had higher survival (35.7%) (Log-Rank, p=0.001), as had those with no NSVT (12.9%) (Log-Rank, p=0.040) (Table 3).

**Table 3:** Comparison of the survival curve and general mortality for the variables analyzed [Log-Hank (Mantel-Cox) - Kaplan-Meier test]

Variables	n	Events	Censored	Survival %	p value
**Age (years)**					0.666
< 60	47	42	5	10.6	
> 60	13	11	2	15.4	
General	60	53	7	11.7	
**SBP (mmHg)**					0.325
< 120	50	45	5	10.0	
>120	10	8	2	20.0	
**Serum sodium (mEq/L)**					0.128
<135	14	13	1	7.1	
135| --- 144	42	37	5	11.9	
> 144	4	3	1	25.0	
**NSVT**					0.040
Yes	29	26	3	10.3	
No	31	27	4	12.9	
**QRS width (ms)**					0.606
Normal (< 120)	18	16	2	11.1	
Extended (>120)	42	37	5	11.9	
**Functional class (NYHA)**					0.066
II	32	26	6	18.8	
III	28	27	1	3.6	
**ILAV (mL/m**^2^**)**					0.001
< 72	14	9	5	35.7	
> 72	46	44	2	4.3	
**Creatinine (mg/dL)**					0.267
> 1.30	16	15	1	6.3	
≤1.30	44	38	6	13.6	
**Ejection fraction (%)**					0.446
>25%	34	30	4	11.8	
≤ 25%	26	23	3	11.5	

SBP: systolic blood pressure; NSVT: non-sustained ventricular tachycardia; ILAV: indexed left atrial volume; ms: millisecond.

### Multivariate analysis - Cox regression

The variables used in Cox regression that remained in the last adjusted model were: NSVT, ILAV, serum sodium, and functional class, but only the first two had significant risk values (Table 4).

**Table 4:** Distribution of the variables based on univariate analysis of risk and Cox regression

Variable	Univariate analysis		Multivariate analysis	
	*Hazard Ratio* 95%CI	Wald coef. (p value)	*Hazard Ratio* 95%CI	Wald coef. (p value)
NSVT	3.0 (1.02 - 8.48)	4.01 (0.045)	3.83 (1.29 - 11.35)	5.84 (0.016)
ILAV	3.4 (1.58 - 7.24)	9.84 (0.002)	3.51 (1.63 - 7.52)	10.77 (0.001)
Sodium	0.9 (0.86 - 1.01)	2.80 (0.095)	0.98 (0.90 - 1.07)	0.22 (0.639)
FC	1.6 (0.96 - 2.86)	3.22 (0.073)	1.34 (0.76 - 2.36)	1.03 (0.311)

NSVT: non-sustained ventricular tachycardia; ILAV: indexed left atrial volume; FC: functional class; CI: confidence interval; coef.: coefficient.

This study identified an increased risk for death of 2.11 (1.04 - 4.31) among patients with NSVT, and of 3.51 (1.63 - 7.52) among those with ILAV ≥ 72 mL/m^2^ (p <0.05 for both).

## Discussion

### Survival in heart failure

In this study, the cumulative overall survival probability of patients with severe Chagasic HF was approximately 11%, resulting from 53 deaths during the 90-month follow-up of a population of 60 patients.

The results found in this study are similar to those by Theodoropoulos et al.,^18^ who have assessed 127 patients with Chagasic HF and found cumulative survival probabilities of 78%, 59%, 46% and 39% in 1-, 2-, 3- and 4-year follow-ups, respectively (Table 5).

**Table 5:** Comparison of cumulative survival rate in the studies

Follow-up [months (years)]	Costa, S.A. (2016)	Theodoropoulos, T.A. et al.^18^	Areosa, C.M.N. et al.^5^	Rassi, S. et al.^19^
	CS (%)	CS (%)	CS (%)	CS (%)
12 (1 year)	70	78	84.5	90.6
24 (2 years)	50	59	74.3	82.3
36 (3 years)	46	46	68.9	73.3
48 (4 years)	40	39	64.8	70.2
60 (5 years)	23	-	60.5	64.4
72 (6 years)	16	-	-	-
84 (7 years)	13	-	-	-
90 (7.5 years)	11	-	-	-

CS: cumulative survival

Clinical studies on HF of different etiologies have shown a slightly better probability in the long run. The cumulative overall survival probabilities reported by Rassi et al.^19^ were 90.6%, 82.3%, 73.3%, 70.2% and 64.4% after 1, 2, 3, 4 and 5 years of follow-up, respectively. That population had HF of recent symptom onset.^19^

The survival reported by Areosa et al.^5^ in a study with patients with severe HF of different etiologies, referred for cardiac transplantation, was 84.5% in the first year, 74.3% in the second year, 68.9% in the third year, 64.8% in the fourth year, and 60.5% in the fifth year.

The patients included in our analysis were properly medicated, and had age groups, functional class, SBP and LVEF similar to those of other studies (Theodoropoulos et al.,^18^ Rassi et a.l^19^ and Areosa et al.^5^). The present study and that by Theodoropoulos et al.^18^ assessed only Chagasic patients, while the other cohorts comprised patients with HF of different etiologies (Table 5), that being their major difference.

Our study follow-up was long (7.5 years). Because Chagasic HF is a severe disease, with high mortality, the survival rate was expected to be low. The comparison of the survival rates reported by Rassi et al.^19^ and Areosa et al.^5^ and ours evidenced the lowest survival rate of severe Chagasic HF since the first year of follow-up, characterizing the worst prognosis of Chagasic individuals. When comparing our results with those by Theodoropoulos et al.,^18^ who recruited only Chagasic patients, the similarity of data is evident.

To our knowledge, ours is the only study following up a population with HF longer than 5 years. Thus, there is no study on a 7.5-year survival that allows the comparison with ours.

#### Prognostic factors with no statistical significance

The variables SBP, age, LVEF, 6MWT, QRS width, and functional class showed no statistical significance regarding the outcome mortality.

Serum sodium and creatinine concentrations showed statistical significance regarding the outcome mortality on univariate analysis; after adjusting the model in multivariate analysis, however, they lost significance.

### Prognostic factors with statistical significance

#### Indexed left atrial volume

This study used the cut-off point of 72 mL/m^2^, similarly to that determined by Rassi et al.,^19^ who identified, by using the ROC curve, 70,71 mL/m^2^ as the best cut-off point.^20^

An ILAV > 72 mL/m^2^ was associated with a significant increase in mortality. Individuals with ILAV > 72 mL/m^2^ had increased risk for death (HR = 3.51; 95% CI: 1.63 - 7.52; p<0.05). Nunes et al.^6^ have assessed the prognostic vale of ILAV in a population of 192 patients with Chagasic HF. They have identified a 4.7% increase in the risk for cardiac events for each 1-mL/m^2^ increment in ILAV (HR = 1.047; 95% CI: 1.035 - 1.059; p <0.001), ILAV being, thus, considered a strong predictor of adverse results, implicating in worse prognosis and increased risk of death in that population.

Of the 20 echocardiographic parameters studied, ILAV proved to be the only independent predictor of cardiovascular mortality in patients with Chagasic HF.^20,21^

The echocardiogram, by identifying ILAV, adds significant information, and is a widely used non-invasive method that can play an important role in risk stratification, follow-up and treatment of Chagasic dilated cardiomyopathy.^10,22^

#### Non-sustained ventricular tachycardia on Holter

The NSVT was one of the variables analyzed with Cox regression that showed significant risk (HR = 2.11; 95% CI: 1.04 - 4.31; p < 0.05). Ventricular arrhythmias, such as NSVT, have been reported as extremely frequent in Chagas disease. The episodes of NSVT have been closely related to the ventricular dysfunction degree and its clinical repercussions, occurring in approximately 40% of the patients with Chagasic HF.^23^

In our case series, all patients had LVEF <35%, and 48.34% of them had NSVT on 24-hour Holter. Despite the high mortality of this population, only 5.67% of the deaths occurred suddenly. These patients were under optimal medical therapy with amiodarone and beta-blocker, which can partially explain this fact.^23^

Two Argentinian randomized studies, GESICA and EPAMSA, assessing the effect of amiodarone in patients with HF, have included 10% and 20% of Chagasic patients in their cohorts, respectively. They have suggested that amiodarone could reduce total mortality when administered to patients with complex ventricular arrhythmias associated with reduced LVEF (< 35%).^23^ However, at the time those studies were conducted, there was no formal indication for the use of beta-blockers in systolic HF.^24^

A sub-analysis of the REMADHE study, assessing the mode of death of patients with Chagasic HF as compared to that of patients with non-Chagasic cardiomyopathy, has shown higher mortality due to progressive HF among Chagasic patients, and that the use of amiodarone in that group was an independent predictor of mortality.^24^

In our case series, no patient had an implantable cardioverter-defibrillator, and 18 (30%) had a pacemaker.

#### Study limitations

This is a retrospective analysis of data prospectively collected in the EMRTCC study, originating from a single center. Despite the limitations inherent in a retrospective analysis, the parameters prospectively collected met well-defined criteria.

In addition, the population studied met very restrictive inclusion criteria, such as functional class (II and III), LVEF (≤ 35%) and creatinine (≤ 2.5mgd/L), which limited the expression of those variables to the correlation analysis with outcomes.

Another limitation was the small number of patients on beta-blockers, which is due to the low blood pressure of that specific population of patients, the bradycardia inherent in the heart disease, added to the use of amiodarone and digitalis.

## Conclusions

In patients with Chagasic HF and important ventricular dysfunction, the presence of NSVT on Holter, as well as an ILAV greater than 72 mL/m^2^ on echocardiography, are independent predictors of mortality.

The general prognosis of those patients is poor, with a cumulative survival probability of 11% in 7.5 years.
